# Minimal important difference and patient acceptable symptom state for PFDI-20 and POPDI-6 in POP surgery

**DOI:** 10.1007/s00192-020-04513-z

**Published:** 2020-09-02

**Authors:** Päivi K. Karjalainen, Nina K. Mattsson, Jyrki T. Jalkanen, Kari Nieminen, Anna-Maija Tolppanen

**Affiliations:** 1 Department of Obstetrics and Gynecology, Central Finland Central Hospital, Keskussairaalantie 19, 40620 Jyväskylä, Finland; 2 University of Eastern Finland, Kuopio, Finland; 3 Department of Obstetrics and Gynecology, Kanta-Häme Central Hospital, Hämeenlinna, Finland; 4 Central Finland Hospital District, Jyväskylä, Finland; 5 Faculty of Medicine and Health Technology, Tampere University, Tampere, Finland; 6 Department of Obstetrics and Gynecology, Tampere University Hospital, Tampere, Finland; 7 School of Pharmacy, University of Eastern Finland, Kuopio, Finland

**Keywords:** Minimal important difference, Patient-acceptable symptom state, Pelvic Floor Distress Inventory-20, Pelvic Organ Prolapse Distress Inventory-6, Pelvic organ prolapse surgery

## Abstract

**Introduction and hypothesis:**

Patient-reported outcome measures are fundamental tools when assessing effectiveness of treatments. The challenge lies in the interpretation: which magnitude of change in score is meaningful for the patients? The minimal important difference (MID) is defined as the smallest difference in score that patients perceive as important. The Patient Acceptable Symptom State (PASS) represents the value of score beyond which patients consider themselves well. We aimed to determine the MID and PASS for Pelvic Floor Distress Inventory-20 (PFDI-20) and Pelvic Organ Prolapse Distress Inventory-6 (POPDI-6) in pelvic organ prolapse (POP) surgery.

**Methods:**

We used data from 2704 POP surgeries from a prospective, population-based cohort. MID was determined with three anchor-based and one distribution-based method. PASS was defined using two different methods. Medians of the estimates were identified.

**Results:**

The MID estimates with (1) mean change, (2) receiver-operating characteristic (ROC) curve, (3) 75th percentile, and (4) distribution-based method varied between 22.9–25.0 (median 24.2) points for PFDI-20 and 9.0–12.5 (median 11.3) for POPDI-6. The PASS cutoffs with (1) 75th percentile and (2) ROC curve method varied between 57.7–62.5 (median 60.0) for PFDI-20 and 16.7–17.7 (median 17.2) for POPDI-6.

**Conclusion:**

A mean difference of 24 points in the PFDI-20 or 11 points in the POPDI-6 can be used as a clinically relevant difference between groups. Postoperative scores ≤ 60 for PFDI-20 and ≤ 17 for POPDI-6 signify acceptable symptom state.

## Introduction

The importance of subjective patient-reported outcome measures (PROMs) in assessing the effectiveness of treatments is widely acknowledged [1, 2]. Typically, these PROMs are questionnaires that measure the burden from various symptoms and yield a continuous score, which can then be used to evaluate differences between groups or change within a group. The challenge lies in the interpretation: which magnitude of difference in score is clinically significant. While statistical significance can be reached in theory in any comparative study by increasing the sample size, the observed difference may be so small that it is not meaningful for patients. To address this challenge, two concepts have been introduced: minimal important difference (MID) and patient acceptable symptom state (PASS).

MID represents the smallest difference in score that patients perceive as important [3, 4]. Two primary approaches, anchor-based and distribution-based, are used to determine the MID. Anchor-based methods correlate the change in the target PROM score with an external criterion, which is typically a single global measure of perceived improvement/deterioration rated by the patient [5]. While anchor-based methods reflect patients’ personal experience, the distribution-based methods use purely mathematical criteria to determine the MID threshold. Due to this lack of external patient-centered reference point, distribution-based methods have been suggested to be used only as supportive evidence or when an anchor-based MID is not available [6]. Among the various anchor-based methods, none have been demonstrated to be superior to the others [5, 6].

While MID is related to change, typically improvement, PASS is used to interpret whether patients have reached sufficient subjective remission of symptoms (= state). PASS represents “the value of score beyond which patients consider themselves well” [7]. MID and PASS are complementary to one another. When patients rate being improved after a treatment, it does not automatically indicate that their state is satisfactory. On the other hand, after only a modest improvement, patients can assess their state as satisfactory in their normal life and may not be willing to pursue further treatment.

The short form of the Pelvic Floor Distress Inventory (PFDI-20) is a condition-specific health-related quality of life instrument measuring a wide range of symptoms related to pelvic floor dysfunction. It consists of three scales: urinary, colorectal/anal, and pelvic organ prolapse (POP). PFDI-20 has been shown to be a valid, reliable, and responsive instrument in both pelvic floor and POP research [8–10].

Three studies have defined MID for PFDI-20 [8, 11, 12]. These studies included patients with diverse pelvic floor disorders and interventions (Appendix Table 4). Consequently, the estimates differed from each other. MID seems to be disease-specific [6]; therefore, studies defining MID in different patient groups are necessary. Accumulating evidence from multiple studies also creates higher confidence in the MID estimate. MID for PFDI-20 delineated specifically for POP surgery has not been previously defined. Furthermore, unlike for the urinary and colorectal scales of the PFDI-20, MID has not been defined for its prolapse-specific scale (Pelvic Organ Prolapse Distress Inventory, POPDI-6).

Until now, the concept of PASS has been more widely studied and used in musculoskeletal research [7, 13]. To the best of our knowledge, there are no previous reports on PASS for PFDI-20.

The aim of our study was to define MID and PASS for PFDI-20 and POPDI-6 in POP surgery.

## Materials and methods

### Study population

We used data from the Finnish Pelvic Organ Prolapse Study (FINPOP). FINPOP is an observational, prospective, nationwide cohort including 3535 POP surgeries performed in Finland in 2015. All Finnish hospitals performing POP surgery were invited to participate and to recruit all patients planned to undergo POP surgery. The cohort includes 83% of the operations performed for POP in the whole country during the study period; 81% (*n* = 2855) of the operations were native tissue repairs, 12% (*n* = 429) were vaginal mesh surgeries, and 7% (*n* = 251) were sacrocolpopexies. The study protocol, population, and methods of surgery have been previously described in detail [14].

### Measurements

The surgeons filled in questionnaires on the patients’ previous gynecological history, degree of prolapse, and details of the surgery at baseline. Participants filled in questionnaires at baseline, 6 months, and 24 months after surgery.

Participants completed PFDI-20 at baseline and at 6 and 24 months after surgery. PFDI-20 consists of three scales: six questions on the inconvenience of POP (POPDI-6), eight questions concerning defecation, and six questions on bladder function. The range of the total score is 0–300 points, and the range for each scale is 0–100, with a higher score indicating higher symptom burden. Missing items are excluded, and the mean from the answered items is used to calculate the total score. The Finnish version of PFDI-20 has been validated [15].

Patients rated their perceived global improvement/deterioration using the Patient Global Impression of Improvement (PGI-I) scale at 6 and 24 months. PGI-I has been validated for use in POP surgery [16]. The wording of the question and choices for answer is as follows: “Check the number that best describes how your postoperative condition is now, compared with how it was before you had the surgery”: (1) very much better; (2) much better; (3) a little better; (4) no change; (5) a little worse; (6) much worse; (7) very much worse.

At 24 months, the participants reported their state by answering the PASS anchor question: “When taking into account your daily activities and your symptoms related to prolapse, do you consider that your state is good enough?” (“yes” or “no”).

### Data handling and statistical analyses

We restricted the analysis to women who had responded to the baseline questionnaire and at least one of the follow-up (6 and/or 24 months) questionnaires (*N* = 2704). We also performed sensitivity analyses on 2623 women after excluding women with concomitant anti-incontinence surgery (*N* = 24) or rectopexy (*N* = 57).

We used baseline and 6 month’s data for MID analysis. We calculated the change of PFDI-20 and POPDI-6 scores for each patient by subtracting the PFDI-20/POPDI-6 score at baseline from the PFDI-20/POPDI-6 score at 6 months. Thus, a negative change score indicated improvement of symptom burden and vice versa.

To assess the usefulness of the MID anchor question, PGI-I, we calculated the Pearson’s correlations between the PGI-I and PFDI-20/POPDI-6 change score and between the PGI-I and PFDI-20/POPDI-6 score at 6 months. We calculated the mean PFDI-20 and POPDI-6 change scores stratified for each PGI-I category.

We determined MID using four previously established methods: three different anchor-based methods—(1) mean change method, (2) receiver-operating characteristics (ROC) curve method, and (3) 75th percentile method—and (4) one distribution-based method: the half a standard deviation (0.5 SD) method. For the anchor-based methods, 7-point PGI-I was used as the anchor.

As per the mean change method, we calculated MID as the mean change in score of women reporting ‘a little better’ in PGI-I minus the mean change in score of women reporting ‘no change’ [6, 17, 18]. As per the ROC curve method, we defined MID as the change score which is associated with the smallest amount of misclassification into improved and not improved according to PGI-I. We included patients reporting ‘a little better,’ ‘much better,’ ‘very much better,’ and ‘no change’ and then dichotomized the patients into improved (‘a little better,’ ‘much better,’ ‘very much better’) and not improved (‘no change’). The MID estimate was determined as the point on the ROC curve maximizing the sum of sensitivity and specificity (the Youden index) [19]. As per the 75th percentile method, we identified the cut-off point corresponding to the 75th percentile of the change score among patients with important improvement (defined as PGI-I answers ‘a little better’ or ‘much better’) [20, 21]. Last, using the distribution-based 0.5 SD method, we took the 0.5 standard deviation (SD) of the baseline mean score as the estimate for MID [22].

To compare the MID estimates with the measurement error, we determined the standard error of measurement (SEM) and smallest detectable change (SDC) in a separate study population. This population was previously used to validate the PFDI-20 in Finnish and has been described in detail by Mattson et al. [15]. Briefly, test-retest measures of PFDI-20 and POPDI-6, assessed at a 2-week interval, were available for 60 and 61 women, respectively. Intraclass correlation coefficient (ICC) for PFDI-20 was 0.92, as reported previously, and SD was 52.4. ICC for POPDI-6 was 0.83, and SD was 5.1. SEM was calculated as SD√(1-ICC). The SDC at the group level was calculated as (1.96xSEMx√2)/√*n* [23]. The SDC at the individual level was calculated as 1.96xSEMx√2 [23].

We used 24 month’s data for PASS analysis. We defined PASS with two previously established methods. Based on the response to the PASS anchor question, the patients were dichotomized into those who had or had not reached PASS (i.e., acceptable state). (1) As per the 75th percentile method, we identified the cut-off point corresponding to the 75th percentile of the 24 months’ score among those reaching PASS [7, 24]. (2) As per the ROC curve method, we plotted the 24 months’ scores against reached/did not reach PASS and then identified the point on the ROC curve that was the best compromise between sensitivity and specificity (= maximized Youden index) [24, 25].

The 95% confidence intervals (CIs) for MIDs and PASSs were derived with bootstrapping based on 1000 replicates.

### Ethical aspects

This study followed the ethical standards for human experimentation established by the Declaration of Helsinki in 1964, revised in 2013. The study was approved by the Research Ethics Committee of the Northern Savo Hospital District (reference number 5//2014), and each participating hospital granted an approval for conducting the study. The study was registered prospectively at ClinicalTrials.gov (NCT02716506). All participants gave written consent. The study was organized and funded by the Finnish Society for Gynecological Surgery, a non-profit organization.

## Results

The study population consists of 2704 patients with the baseline questionnaire and at least one of the follow-up questionnaires available (6 months *N* = 2535, 24 months *N* = 2349). Of them, 24 (1%) underwent concomitant anti-incontinence surgery (mid-urethral sling), and 57 (2%) underwent concomitant rectopexy. The characteristics of the study population are presented in Table 1.

**Table 1:** Characteristics of the study population (*n* = 2704)

Characteristic	Value
Age, years, mean ± SD	64.2 ± 10.3
BMI, kg/m², mean ± SD	26.9 ± 4.0
Parity, median (IQR)	2 (1)
Prior hysterectomy, *n* (%)	916 (33.9)
Prior prolapse surgery, *n* (%)	683 (25.3)
Prior anti-incontinence surgery, *n* (%)	157 (5.8)
Current smoker, *n* (%)	219 (8.1)
POP-Q point Ba ≥ 0, *n* (%)	1714 (65.5)
POP-Q point Bp ≥ 0, *n* (%)	1158 (44.4)
POP-Q point C ≥ 0, *n* (%)	1047 (40.5)
PFDI-20 baseline score ^a^, mean ± SD	98.8 ± 49.8
POPDI-6 baseline score ^b^, mean ± SD	40.8 ± 20.2

^a^The scale of the score is 0–300, higher score indicating higher symptom burden

^b^The scale of the score is 0–100, higher score indicating higher symptom burden

*SD* standard deviation, *BMI* body mass index, *POP-Q* Pelvic Organ Prolapse Quantification, *PFDI-20* Pelvic Floor Distress Inventory-20, *POPDI-6* Pelvic Organ Prolapse Distress Inventory-6

### Minimal important difference for PFDI-20 and POPDI-6

PGI-I and PFDI-20 change score at 6 months correlated moderately (*r* = 0.33; *p* < 0.001). The correlation between PGI-I and 6 month’s postoperative PFDI-20 score was strong (*r* = 0.51, *p* < 0.001). PGI-I and POPDI-6 change score at 6 months correlated moderately (*r* = 0.35; *p* < 0.001). The correlation between PGI-I and 6 month’s postoperative POPDI-6 score was strong (*r* = 0.53, *p* < 0.001). Table 2 presents the PFDI-20 and POPDI-6 change between baseline and 6 months for each PGI-I category.

**Table 2:** Mean PFDI-20 and POPDI-6 change scores for each global impression of change category

Patient Global Impression of Improvement	*n* = 2475 ^a^	PFDI-20 change score ^b^	POPDI-6 change score ^c^
	*p* (%)	Mean (95% CI)	Mean (95% CI)
Very much better	842 (34.0)	−71.8 (−74.8 to −68.8)	−37.5 (−38.9 to −36.1)
Much better	1133 (45.8)	−55.7 (−58.1 to −53.3)	−30.0 (−31.1 to −28.9)
A little better	335 (13.5)	−37.9 (−42.5 to −33.4)	−19.4 (−21.3 to −17.4)
No change	95 (3.8)	−13.5 (−21.9 to −5.1)	−10.4 (−14.9 to −6.0)
A little worse	36 (1.5)	−30.5 (−48.5 to −12.6)	−14.2 (−22.1 to −6.3)
Much worse	28 (1.1)	−8.5 (−26.2 to 9.1)	−9.9 (−19.2 to −0.6)
Very much worse	6 (0.2)	−16.8 (−54.8 to 21.2)	−5.6 (−25.8 to 14.7)
All	2475 (100)	−56.2 (−57.9 to −54.4)	−29.8 (−30.7 to −29.0)

^a^Calculated for patients with PFDI-20 change score, POPDI-6 change score, and Patient Global Impression of Improvement available at 6 months

^b^The scale of the score is 0–300, higher score indicating higher symptom burden. Negative value in change score indicates improvement. ^c^The scale of the score is 0–100, higher score indicating higher symptom burden. Negative value in change score indicates improvement

*PFDI-20* Pelvic Floor Distress Inventory-20, *POPDI-6 CI* Pelvic Organ Prolapse Distress Inventory-6, *CI* confidence interval

**Fig. 1:**
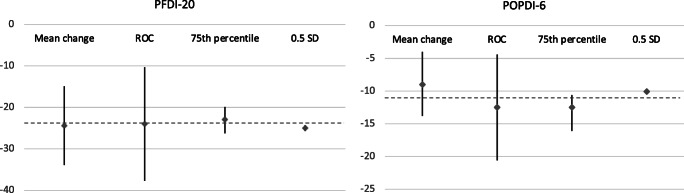
MID for PFDI-20 and POPDI-6 derived with anchor-based and distribution-based methods. MID estimates defined with four different methods and their 95% confidence intervals. Vertical lines denote 95% confidence intervals. The dashed line indicates the median: - 24 points for PFDI-20 and -11 points for POPDI-6. MID, minimal important difference; PFDI-20, Pelvic Floor Distress Inventory-20; POPDI-6, Pelvic Organ Prolapse Distress Inventory-6; ROC, receiver-operating characteristics; SD, standard deviation

The MID estimates (for improvement) for PFDI-20 were as follows: (1) mean change method, −24.4 (95% CI -33.9 to −14.9); (2) ROC curve method, −24.0 (95% CI -37.7 to −10.3) [area under the curve (AUC) 0.79 (95% CI 0.75 to 0.84)]; (3) 75th percentile method −22.9 (95% CI -26.2 to −19.9); 4) 0.5 SD method, −25.0 (95% CI -25.7 to −24.3). The median of the estimates was −24.2. (Fig. 1).

The MID estimates (for improvement) for POPDI-6 were as follows: (1) mean change method, −9.0 (95% CI -13.8 to −4.0); (2) ROC curve method, −12.5 (95% CI -20.6 to −4.4) [AUC 0.76 (95% CI 0.71 to 0.81)]; (3) 75th percentile method, −12.5 (95% CI -16.1 to −10.6); (4) 0.5 SD method, −10.1 (95% CI -10.4 to −9.9). The median of the estimates was −11.3. (Fig. 1).

SEM for PFDI-20 was 14.8. The SDC at the group level was 5.3. The SDC at the individual level was 41.1. The SEM for POPDI-6 was 2.1. The SDC at the group level was 0.75. The SDC at the individual level was 5.8.

### Patient-acceptable symptom state for PFDI-20 and POPDI-6

At 24 months, 84% of the patients reported having reached PASS. The proportion of patients reaching PASS for each PGI-I category is given in Table 3. The mean PFDI-20 score at 24 months among those reaching PASS was 38.4 (95% CI 36.8 to 39.9) and for those not reaching PASS 103.2 (95% CI 97.7 to 108.6). The mean POPDI-6 score at 24 months among those reaching PASS was 9.2 (95% CI 8.7 to 9.7) and for those not reaching PASS 33.9 (95% CI 31.7 to 36.1).

**Table 3:** Proportion of patients reaching patient acceptable symptom state (PASS) at 2 years' follow-up for each global impression of change category

PGI-I	Patients reporting to have reached PASS ^a^ N reaching PASS/N patients per PGI-I group	%; 95% CI
Very much better	660/665	99; 98 to 100
Much better	945/990	96; 94 to 97
A little better	233/381	61; 56 to 66
No change	40/116	35; 26 to 44
A little worse	14/58	24; 14 to 37
Much worse	4/33	12; 3 to 28
Very much worse	6/18	33; 13 to 59
All	1902/2261	84; 82 to 85

^a^Calculated for patients with Patient Global Impression of Improvement and Patient Acceptable Symptom State available at 24 months

*PASS* Patient Acceptable Symptom State, *PGI-I* Patient Global Impression of Improvement, *CI* confidence interval

The PASS estimates for PFDI-20 were as follows: (1) 75th percentile method 57.5 points (95% CI 54.9 to 60.4) and (2) ROC curve method 62.5 [95% CI 41.4 to 83.6; sensitivity 78%, specificity 78%; AUC 0.87 (95% CI 0.85 to 0.88)]. The median of the PASS estimates for PFDI-20 was 60.0.

The PASS estimates for POPDI-6 were as follows: (1) 75th percentile method 16.7 (95% CI 12.6 to 18.8) and (2) ROC curve method 17.7 [95% CI 13.1 to 22.3; sensitivity 73%, specificity 84%; AUC 0.86 (95% CI 0.83 to 0.88)]. The median of the PASS estimates for POPDI-6 was 17.2.

The sensitivity analyses excluding the women undergoing concomitant anti-incontinence surgery or rectopexy yielded similar MID and PASS estimates (Appendix Table 5).

## Discussion

Our large, population-based study on women undergoing POP surgery showed that a reduction of 24 points in PFDI-20 score and a reduction of 11 points in POPDI-6 score indicate a clinically meaningful improvement within a group or a clinically relevant difference between groups. We used four different methods to define MID, and all methods produced consistent estimates. In addition to MID, we defined PASS estimates for PFDI-20 and POPDI-6. According to our results, a postoperative PFDI-20 score of 60 and a postoperative POPDI-6 score of 17 can be used as a cut-off below which patients are likely to have reached an acceptable state in terms of their symptoms.

Our results complement the three previous reports on MID for PFDI-20 with MID estimates varying between 13.5 and 45 points [8, 11, 12] (Appendix Table 4). None of these studies estimated MID specifically in a POP surgery population. Since MID may vary across clinical conditions [6], it is not reasonable to expect that a single MID would be applicable in all populations. Wiegersma et al. estimated a MID of 13.5 points among women undergoing *conservative* treatment for POP. The lower MID estimate in their study was unsurprising. Their population had a lower baseline score (56 points) than our population (99 points) likely related to the fact that women opt for surgical treatment when the symptom burden is high. Several studies have shown that the MID is dependent on the baseline score, with a higher symptom burden requiring a higher change to be perceived [20, 26]. The studies by Barber et al. (MID 45 points) and by Utomo et al. (23 points) comprised populations with *any* pelvic floor dysfunction. The first included women undergoing surgical treatment, the latter both conservative and surgical treatment. The discrepancy with the estimate of the Barber et al. study may be because they did not subtract the mean change score of the ‘no change’ group as there were no women who reported ‘no change.’

To the best of our knowledge, no previous reports on MID for POPDI-6 exist in the literature. Barber et al. showed that the subscales of PFDI were the most responsive to the respective pelvic floor disorder of primary interest, i.e., responsiveness for the POPDI was the highest when the study population was POP patients and lowest when POP was not the primary condition [9]. POP patients commonly present with various pelvic floor symptoms; thus, it seems sensible to use both PFDI and POPDI in women with POP.

The methods to define MID and PASS are not yet standardized [13]. Open questions include, but are not limited to: the preferred statistical method or combination of methods, wording of the anchors, cut-off in the anchor (a little better for minimal difference or much better for important difference), follow-up time, and adjustment for confounders such as the baseline score. As there is no clear agreement on the best method to define MID and PASS at present, we used multiple methods and provided the medians of the estimates. The estimates for both MID and PASS obtained with different methods were relatively similar, allowing us to select the median as the proposed MID/PASS value.

A recent paper by Devji et at. provides an instrument to critically evaluate the quality of the available MID [27]. Five core items in a credible MID are: anchor and PROM answered by the patients themselves; anchor easily understandable for the patients; good correlation between the anchor and the PROM; precise MID estimate (narrow confidence intervals or large sample); threshold on the anchor reflects a small but important difference (rather than moderate or large). Four of these criteria are met in our study, but the correlation between the anchor and the PROM is suboptimal. The correlation between the anchor and change score in our study was 0.32 for PFDI-20 and 0.35 for POPDI-6. A correlation threshold of 0.30–0.35 for a credible MID is often quoted [6]. However, some authorities have suggested a threshold as high as 0.5 or 0.7 [27, 28]. The anchor correlated more strongly with the postoperative score than with the change score. This phenomenon has been noted previously as well and reflects the shortcoming of the global transition rating as an anchor [29]. It seems that patients are biased by their current state at the time of rating and cannot recall their preoperative state to which they should make a comparison. On the other hand, while PFDI-20 attempts to capture a comprehensive picture of the pelvic floor function, it may fail to capture the individual perspective. For example, a woman may see an improvement in bulge and bladder storage symptoms after POP surgery, but may experience bothersome de novo stress urinary incontinence or dyspareunia (the latter not measured by PFDI-20). She may perceive her state as much worse than before the surgery even though her score improved markedly.

Our MID estimates for PFDI-20 and POPDI-6 can distinguish clinically important change from measurement error with high certainty when used *at the group level*. However, because measurement error (as indicated by SDC) *at the individual level* is larger than the MID, PFDI-20 is not suited to follow up individuals in clinical practice, there is a considerable chance that an observed change of the size of 24 points (= MID) is due to measurement error.

MID and PASS can be used concurrently in interpretation of PROM scores in comparative and observational studies. The principle role of MID is to interpret group-level mean differences: if a statistically significant difference in change score between groups is greater than the MID, it can be interpreted as a clinically meaningful difference in the efficacy. Second, MID and PASS can be used in responder analysis to report or to compare how large a proportion of patients experienced a meaningful improvement and, perhaps more importantly, reached an acceptable state. Since the difference in the PROM score may be difficult to grasp, the proportion of responders provides a useful tool for clinical decision-making and patient counseling. Third, MID can be utilized in sample size calculations to signify the smallest difference the study needs to detect.

We suggest adding the concept of PASS into the armamentarium of gynecological clinical research. PASS may be a more relevant measure for patients compared to MID—after reaching an improvement comparable to MID, one can still suffer from symptom burden that is beyond subjective tolerance. Furthermore, unlike MID, PASS can be used to compare trial results when the baseline PROM score is not available.

### Strengths and limitations

The strengths of our study include: a large, population-based sample and wide diversity of surgical methods increase the generalizability of the results; multiple statistical methods yielding consistent MID estimates suggest robustness of the results.

The most important limitation of our study is that the correlation between the anchor (PGI-I) and the PROM change score was only moderate. However, the MIDs detected by the distribution-based method were nearly equal to those detected with the anchor-based methods. Another limitation is that the small number of patients with no change/deterioration prevented us from estimating the MID for deterioration.

### Conclusions

We provided MID and PASS estimates to aid the interpretation of PFDI-20 and POPDI-6 scores in POP surgery. A mean difference of 24 points in the PFDI-20 score and 11 points in the POPDI-6 score can be used as a clinically relevant difference between groups. A postoperative PFDI-20 score ≤ 60 and a POPDI-6 score ≤ 17 can be used in responder analysis (acceptable state).

## Appendix

**Table 4:** Characteristics of previous studies estimating the minimal important difference (for improvement) for Pelvic Floor Distress Inventory-20

Author	Year	N	Population	Method to determine MID	Anchor	MID
Barber [8]	2005	45	Surgery for any pelvic floor dysfunction; POP 58%; baseline score 122; follow-up 3–6 months	Anchor based; mean change in score of those being ‘a little better’ on the global rating scale (*N* = 7)	Global perception of improvement; 7 points	45
Utomo [12]	2014	67	Urinary incontinence, POP (57%), fecal incontinence; Conservative, pharmaceutical, or surgical treatment (66%); baseline score 94; follow-up 6 months	Anchor based; ROC-curve method Question 2 in the RAND 36 was dichotomized: patients reporting to be “a little better” or “much better” were classified as “improved,” while “same,” “a little worse,” or “much worse” were classified as “not improved”	RAND 36; question 2, rating of patient’s impression of their general health compared to 1 year ago; 5-point	23
Wiegersma [11]	2017	214	Conservative treatment for POP; baseline score 56; follow-up 12 months	Anchor based; ROC curve method; global perception of improvement was dichotomized to categories “better” and “much better” vs “about the same,” “worse”, and “much worse”	Global perception of improvement; 5-point	13.5

*MID* minimal important difference, *POP* pelvic organ prolapse, *TVM* transvaginal mesh, *MUS* mid-urethral sling, *ROC* receiver-operating characteristic

**Table 5:** MID and PASS for PFDI-20 and POPDI-6 excluding patients with concomitant anti-incontinence surgery or rectopexy *N* = 2623

Method used	MID for PFDI-20 (95% CI)	MID for POPDI-6 (95% CI)	PASS for PFDI-20 (95% CI)	PASS for POPDI-6 (95% CI)
Mean change	−24.1 (−33.9 to −14.9)	−8.7 (−13.8 to −4.0)	NA	NA
ROC curve	−24.0 (−38.7 to −9.2)	−12.5 (−20.1 to −4.9)	57.3 (54.4 to 60.1)	16.7 (11.7 to 18.8)
75th percentile	−22.1 (−26.2 to −19.9)	−12.5 (−16.1 to −10.6)	62.5 (47.9 to 85.4)	17.7 (12.1 to 23.4)
0.5 SD	−24.7 (−25.7 to −24.3)	−10.1 (−10.4 to −9.9)	NA	NA
Median for above estimates	−24.1	−11.3	59.9	17.2

MID and PASS estimates defined with different methods and their medians. Sensitivity analyses excluding women with concomitant anti-incontinence surgery (*N* = 24) and rectopexy (*N* = 57)

*MID* minimal important difference, *PFDI-20* Pelvic Floor Distress Inventory-20, *CI* confidence interval, *POPDI-6* Pelvic Organ Prolapse Distress Inventory-6, *PASS* Patient Acceptable Symptom State, *ROC* receiver-operating characteristics, *SD* standard deviation, *NA* not applicable

## Abbreviations

PROM: Patient-reported outcome measure
MID: Minimal important difference
PASS: Patient-acceptable symptom state
PFDI-20: Pelvic Floor Distress Inventory-20
POP: Pelvic organ prolapse
POPDI-6: Pelvic Organ Prolapse Distress Inventory-6
ROC: Receiver-operating characteristics
SD: Standard deviation
SEM: Standard error of measurement
SDC: Smallest detectable change
ICC: Intraclass correlation coefficient
CI: Confidence interval
AUC: Area under the curve
